# Comparison of the endoscopic thyroidectomy *via* areola approach and open thyroidectomy: A propensity score matched cohort study of 302 patients in the treatment of papillary thyroid non-microcarcinoma

**DOI:** 10.3389/fonc.2023.1081835

**Published:** 2023-02-28

**Authors:** Yujun Li, Zhaodi Liu, Zhuolin Song, Yong Wang, Xing Yu, Ping Wang

**Affiliations:** Department of Thyroid Surgery, The Second Afﬁliated Hospital of Zhejiang University, School of Medicine, Hangzhou, Zhejiang, China

**Keywords:** papillary thyroid non-microcarcinoma, endoscopic thyroidectomy *via* areola approach, safety, feasibility, cosmetic outcome

## Abstract

**Background:**

The endoscopic thyroidectomy *via* areola approach (ETAA) is widely used in patients with benign thyroid tumors and papillary thyroid microcarcinoma (PTMC). Its safety and complication rates are reported to be similar to open thyroidectomy (OT). This study aimed to evaluate the safety and feasibility of ETAA, compared with OT, in patients with papillary thyroid non-microcarcinoma (PTNMC).

**Methods:**

We retrospectively reviewed all patients with PTNMC who underwent ETAA or OT in our hospital from January 2017 to December 2021. A total of 302 patients were matched at a ratio of 1:1 by the propensity score matching (PSM) analysis and surgical outcomes. Safety and feasibility were analyzed between two groups.

**Results:**

Before PSM, patients in the ETAA group were younger (*p* < 0.001) and had a larger proportion of female patients (*p* < 0.001) with a lower BMI (*p* < 0.001) compared with the OT group. The ETAA group also had a higher proportion of unilateral thyroidectomy (*p* = 0.002). PSM was used to create a highly comparable control group. After PSM, the ETAA group had a longer operative time (*p* < 0.001), larger blood loss (*p* = 0.046) and total drainage amount (*p* = 0.035), with higher C-reactive protein (*p* = 0.023) and better cosmetic outcomes (*p* < 0.001). There were no significant differences in the following clinicopathologic characteristics: number of dissected positive lymph nodes, rate of recurrent laryngeal nerve signal weakened, parathyroid autotransplantation, postoperative pain, hospital stay, complications, and oncologic completeness. There was no patient converted to OT in the ETAA group and two patients suffered from persistence/recurrence in the follow-up.

**Conclusion:**

ETAA is a safe and feasible surgical approach for patients with PTNMC.

## Introduction

Papillary thyroid carcinoma (PTC) has an excellent prognosis and is much more common in young women, who usually pay attention not only to surgical thoroughness and safety but also to cosmetic requirements (1). The endoscopic technique, first reported by Gagner (2), has rapidly advanced over the past two decades. It significantly improves the cosmetic outcomes and quality of life (QoL) by making incisions not from the neck but to hidden areas of the body (3). At present, the endoscopic thyroidectomy *via* areola approach (ETAA) is the most common surgical approach in China. ETAA is widely used in patients with benign thyroid tumors and papillary thyroid microcarcinoma (PTMC, defined as PTC with a diameter ≤1 cm). Its safety and complication rates are reported to be similar to open thyroidectomy (OT) (4, 5).

Although the treatment strategy for PTMC remains controversial (6), according to the 2015 American Thyroid Association (ATA) guidelines, surgery is necessary for patients with papillary thyroid non-microcarcinoma (PTNMC, defined as PTC with a diameter >1 cm) (7). However, few studies have reported the surgical outcomes and safety of ETAA in the treatment of PTNMC to date. Thus, we conduct this study to evaluate the safety and feasibility of ETAA in patients with PTNMC, compared with OT.

## Materials and methods

We retrospectively reviewed 6,469 patients with PTNMC who underwent ETAA or OT from January 2017 to December 2021 in Jiefang Road District, Second Affiliated Hospital of Zhejiang University, School of Medicine. This study has been approved by the Ethical Committee of the Second Affiliated Hospital of Zhejiang University’s School of Medicine.

Data from patients were collected and divided into four categories: (1) clinicopathologic characteristics including age, sex, body mass index (BMI), tumor size, Hashimoto’s thyroiditis, multifocality, and extent of surgery; (2) intraoperative outcomes including operative time, blood loss, and number of dissected and positive lymph nodes; (3) postoperative outcomes such as 24-h visual analog scale (VAS), total drainage amount, white blood cell amount, C-reactive protein level, and complications; and (4) follow-up outcomes such as cosmetic satisfaction, scar self-consciousness, QoL, oncologic completeness, and recurrence.

**Figure 1:**
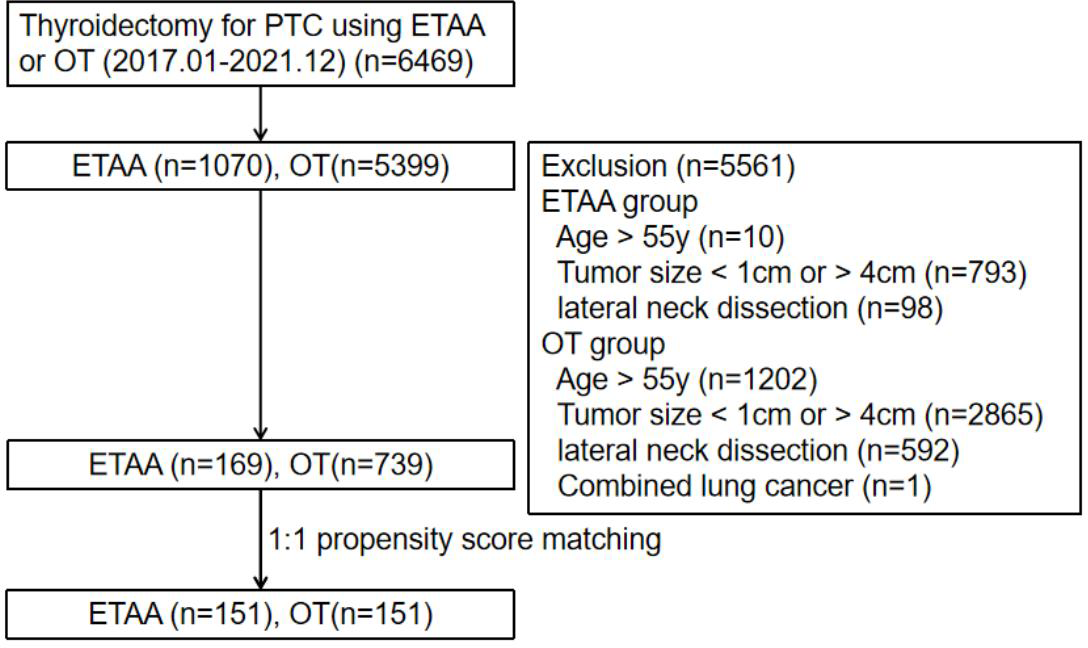
Study flowchart. ETAA, endoscopic thyroidectomy *via* areola approach; OT, open thyroidectomy; PTC, papillary thyroid carcinoma.

According to national consensus and our previous study (8), the inclusion criteria were as follows: (1) PTC diagnosed by postoperative pathological results; (2) age range from 18 to 55 years; (3) BMI range from 18 to 28 kg/m^2^ (9); and (4) no previous history of neck radiation therapy or surgery. The exclusion criteria were as follows: (1) age ≥55 years; (2) tumor maximum diameter evaluated by preoperative ultrasound was <1 cm or >4 cm (10); (3) extrathyroidal or capsular invasion; and (4) cervical lateral lymph node or distant metastasis. According to the inclusion and exclusion criteria, 169 patients in the ETAA group and 739 patients in the OT group were finally included. Then, the propensity score matching (PSM) analysis (11) was conducted to match 151 patient pairs to reduce the potential confounding of clinicopathologic characteristics (**Figure 1**).

Surgical procedures of ETAA (12) and OT (13) were described in detail in our previous article. Tumors with more than two lesions were defined as multifocal. Postoperative vocal cord paralysis, confirmed by laryngoscopy, which recovered within 6 months, was defined as transient recurrent laryngeal nerve (RLN) injury. Transient hypoparathyroidism is diagnosed when the serum parathyroid hormone is smaller than 15 pg/ml. Other complications have been defined in a previous study (8). Total complication referred to the total number of patients with postoperative complications. The follow-up duration was defined as the time between the patient’s surgery day and telephone return visit in March 2022.

According to the recommended follow-up strategy in the ATA guidelines, all patients should undergo serum thyroid function testing and ultrasonography to monitor recurrence every 3 or 6 months. For patients who underwent bilateral thyroidectomy, the serum stimulated thyroglobulin (sTg) level and the percentage of sTg below 1 μg/L were evaluated 3 weeks after discharge and followed by radioactive iodine (RAI) therapy if necessary according to the guidelines. In addition, a questionnaire regarding cosmetic satisfaction, scar self-consciousness, and QoL was sent out 3 months after surgery. Cosmetic satisfaction (14) and scar selfconsciousness (15) were scaled with scores from 0 to 3, with 3 meaning very satisfied and very concerned, respectively. QoL (16) was evaluated with scores ranging from 0 (worst) to 10 (best).

### Statistics

Data were analyzed *via* the statistical program SPSS version 26 (SPSS^®^ Inc., Illinois, USA). Continuous variables were compared using independent-samples *t*-test or Mann–Whitney *U* test, presented as medians ± standard deviations. Categorical variables were analyzed by the chi-squared test or Fisher’s exact test, expressed as numbers and percentages. A *p*-value of <0.05 indicated statistical significance.

## Results

### Clinicopathologic characteristics before and after PSM

The clinicopathologic characteristics are presented in **Table 1**. Before PSM, 169 patients were included in the ETAA group, and 739 patients were included in the OT group. Compared with the OT group, patients in the ETAA group were younger (32.33 ± 7.72 years *vs*. 40.89 ± 9.18 years, *p* < 0.001) and had a larger proportion of female patients (88.7% *vs*. 61.4%, *p* < 0.001). Also, the ETAA group had a lower BMI (21.94 ± 2.96 kg/m^2^ *vs*. 24.16 ± 3.60 kg/m^2^, *p* < 0.001) and a higher proportion of unilateral thyroidectomy (73.8% *vs*. 60.9%, *p* = 0.002). However, there were no statistically significant differences in tumor size (1.43 ± 0.50 cm *vs*. 1.44 ± 0.43 cm, *p* = 0.103), rate of Hashimoto’s thyroiditis (39.9% *vs*. 38.0%, *p* = 0.655), or multifocality (22.6% *vs*. 28.1%, *p* = 0.146). PSM matched a total of 151 patient pairs for subsequent analysis and there were no statically significant differences among the seven clinicopathologic characteristics mentioned above between the two groups.

**Table 1:** Clinicopathologic characteristics.

Variables	Before PSM			After PSM		
	ETAA (*n* = 169)	OT (*n* = 739)	*p*	ETAA (*n* = 151)	OT (*n* = 151)	*p*
Age (years)	32.33 ± 7.72	40.89 ± 9.18	<0.001	33.11 ± 7.52	34.29 ± 7.54	0.928
Sex (*n*, %)						
Male	19 (11.3)	285 (38.6)	<0.001	18 (11.9)	18 (11.9)	1.000
Female	149 (88.7)	454 (61.4)		133 (88.1)	133 (88.1)	
BMI (kg/m2)	21.94 ± 2.96	24.16 ± 3.60	<0.001	22.15 ± 2.96	22.03 ± 2.99	0.577
Tumor size (cm)	1.43 ± 0.50	1.44 ± 0.43	0.103	1.44 ± 0.51	1.46 ± 0.44	0.484
Hashimoto’s thyroiditis (*n*, %)	67 (39.9)	281 (38.0)	0.655	61 (40.4)	66 (63.5)	0.560
Multifocality (*n*, %)	38 (22.6)	208 (28.1)	0.146	34 (38.5)	43 (28.5)	0.235
Extent of surgery (*n*, %)						
Unilateral thyroidectomy	124 (73.8)	450 (60.9)	0.002	110 (72.8)	96 (63.6)	0.084
Bilateral thyroidectomy	44 (26.2)	289 (39.1)		41 (27.2)	55 (36.4)	

BMI, body mass index; PSM, propensity score matching; ETAA, endoscopic thyroidectomy via areola approach; OT, open thyroidectomy.

### Intraoperative outcomes

The intraoperative outcomes are presented in **Table 2**. Compared with the OT group, patients in the ETAA group had a longer operative time (165.42 ± 52.26 min *vs*. 84.47 ± 28.18 min, *p* < 0.001) and a larger blood loss (14.96 ± 5.59 ml *vs*. 14.42 ± 3.86 ml, *p* = 0.046). However, the results of the number of dissected lymph nodes (8.25 ± 6.01 *vs*. 10.87 ± 6.43, *p* = 0.196), positive lymph nodes (2.86 ± 2.33 *vs*. 3.52 ± 2.57, *p* = 0.288), the rate of metastatic lymph nodes (57.6% *vs*. 65.6, *p* = 0.156), weakened RLN signal weaken (7.9% *vs*. 9.9%, *p* = 0.545), and parathyroid autotransplantation results (43.0% *vs*. 40.4%, *p* = 0.641) were similar. No patients were converted to OT in the ETAA group.

**Table 2:** Intraoperative outcomes.

Variables	ETAA (*n* = 151)	OT (*n* = 151)	*p*
Operative time (min)	165.42 ± 52.26	84.47 ± 28.18	<0.001
Blood loss (ml)	14.96 ± 5.59	14.42 ± 3.86	0.046
Number of dissected lymph nodes (piece)	8.25 ± 6.01	10.87 ± 6.43	0.196
Positive (*n*, %)	87 (57.6)	99 (65.6)	0.156
Negative (*n*, %)	64 (42.4)	52 (34.4)	
Number of positive lymph nodes (piece)	2.86 ± 2.33	3.52 ± 2.57	0.288
Rate of RLN signal weaken (*n*, %)	12 (7.9)	15 (9.9)	0.545
Rate of parathyroid autotransplantation (*n*, %)	65 (43.0)	61 (40.4)	0.641
Conversion to open	0	NA	NA

RLN, recurrent laryngeal nerve; ETAA, endoscopic thyroidectomy via areola approach; OT, open thyroidectomy.

### Postoperative outcomes

The postoperative outcomes are presented in **Table 3**. The C-reactive protein (9.58 ± 6.99 mg/L *vs*. 6.29 ± 5.55 mg/L, *p* = 0.023) and total drainage amount (157.65 ± 74.93 ml *vs*. 121.51 ± 74.91 ml, *p* = 0.035) are higher in the ETAA group than in the OT group. There were no statistically significant differences between the two groups in the 24-h VAS (1.76 ± 0.46 *vs*. 1.72 ± 0.51, *p* = 0.285), WBC amount (9.17 ± 2.71 × 10^9^/L *vs*. 9.18 ± 5.59 × 10^9^/L, *p* = 0.598), drainage duration (3.16 ± 1.33 days *vs*. 3.56 ± 1.31 days, *p* = 0.338), postoperative hospital stay (3.20 ± 1.27 days *vs*. 3.60 ± 1.29 days, *p* = 0.572), and proportion of total complications (15.9% *vs*. 18.5%, *p* = 0.542). None of the patients suffered from permanent RLN palsy or hypoparathyroidism.

**Table 3:** Postoperative outcomes.

Variables	ETAA (*n* = 151)	OT (*n* = 151)	*p*
24-h VAS (score)	1.76 ± 0.46	1.72 ± 0.51	0.285
WBC amount (109/L)	9.17 ± 2.71	9.18 ± 5.59	0.598
C-reactive protein (mg/L)	9.58 ± 6.99	6.29 ± 5.55	0.023
Drainage duration (days)	3.16 ± 1.33	3.56 ± 1.31	0.338
Total drainage amount (ml)	157.65 ± 74.93	121.51 ± 74.91	0.035
Postoperative hospital stay (days)	3.20 ± 1.27	3.60 ± 1.29	0.572
Complication (*n*)			
Total	24 (15.9)	28 (18.5)	0.542
Transient RLN palsy	4	6	0.750
Permanent RLN palsy	0	0	NA
Transient hypoparathyroidism	19	23	0.430
Permanent hypoparathyroidism	0	0	NA
Chyle fistula	2	5	0.448
Choking cough	0	1	1.000
Infection	0	0	NA
Hematoma	0	0	NA

VAS, visual analog scale; WBC, white blood cells; RLN, recurrent laryngeal nerve; ETAA, endoscopic thyroidectomy via areola approach; OT, open thyroidectomy.

### Follow-up outcomes

The follow-up outcomes are presented in **Table 4**. The patients had a higher QoL (9.58 ± 6.99 *vs*. 6.29 ± 5.55, *p* < 0.001) in the ETAA group than in the OT group, resulting from the higher cosmetic satisfaction (2.42 ± 0.62 *vs*. 1.55 ± 0.71, *p* < 0.001) and lower scar self-consciousness (0.74 ± 0.64 *vs*. 1.30 ± 0.53, *p* < 0.001). As for oncologic completeness in bilateral thyroidectomy, the stimulated Tg level before RAI (0.89 ± 2.26 μg/L *vs*. 1.17 ± 2.69 μg/L, *p* = 0.597), the rate of Tg level <1 μg/L (65.9% *vs*. 60.0%, *p*#146;= 0.404), and receiving RAI (22.0% *vs*. 25.5%, *p* = 0.740) were not statistically different. During the follow-up, two patients in the ETAA group and one patient in the OT group suffered from persistence/recurrence and underwent a second operation, which showed no significant difference between two groups (*p* = 0.442).

**Table 4:** Follow-up outcomes.

Variables	ETAA (*n* = 151)	OT (*n* = 151)	*p*
Cosmetic satisfaction (score)	2.42 ± 0.62	1.55 ± 0.71	<0.001
Scar self-consciousness (score)	0.74 ± 0.64	1.30 ± 0.53	<0.001
Quality of life (score)	8.50 ± 0.85	7.76 ± 0.84	<0.001
Oncologic completeness			
In bilateral thyroidectomy			
Stimulated Tg level before RAI (μg/L)	0.89 ± 2.26	1.17 ± 2.69	0.597
<1 (*n*, %)	27/41 (65.9)	33/55 (60.0)	0.404
≥1 (*n*, %)	14/41 (34.1)	22/55 (40.0)	
Rate of receiving RAI (*n*, %)	9/41 (22.0)	14/55 (25.5)	0.740
Persistence/Recurrence (*n*, %)	2 (1.3)	1 (0.7)	0.442
Follow-up duration (months)	37.62 ± 14.93	30.60 ± 16.61	0.121

Tg, thyroglobulin; RAI, radioactive iodine; ETAA, endoscopic thyroidectomy via areola approach; OT, open thyroidectomy.

## Discussion

With the development of endoscopic technology and surgical instruments, various approaches such as transaxillary, transareola, bilateral axillo-breast (BABA), and transoral surgery have been designed and new clinical procedures have been rapidly advancing (17). Each approach has its own advantages and disadvantages. ETAA has its unique strengths (18). First, the surgical procedure is well researched and widely used in China. The surgical visual angle, similar to OT, is beneficial for the bilateral thyroidectomy and dissection of the prelaryngeal and lateral neck lymph nodes. Second, all of three cambered incisions are short and hidden, which will have excellent cosmetic results after healing. Third, larger operative space means less mutual interference of surgical instruments. Additionally, it has great clinical benefits and safety in PTMC, which have been confirmed by multiple independent studies (4, 5, 18). The lack of study on PTNMC presents a challenge in the expansion of indications for ETAA. Therefore, we conducted this study to evaluate the safety and feasibility of ETAA in the treatment of PTNMC.

In our center, complete removal of tumor with a maximum diameter > 4 cm was quite a challenge despite lengthening the incision and widening the main tunnel; thus, only T1 and T2 PTNMC were included in our study (10).

Similar to our previous study (19), the ETAA group had a longer operative time and a larger blood loss, which were mainly related to the establishment of surgical space and extensive separation of the thoracic flaps. It was acceptable because the difference in blood loss did not result in changes in vital signs and hospital stay. Yang et al. (20) reported a non-visual method for establishing the operative space to reduce the operative time and separative flap area at the expense of improving surgical difficulties. In addition, experience proved to be beneficial in shortening surgical time (21).

The incidence of complications is vital to assess the safety of endoscopic thyroid surgery. The major complications include RLN injury, hypoparathyroidism, superior laryngeal nerve (SLN) injury, infection, and hematoma, which did not vary significantly between the two groups. The incidence of transient RLN injury ranged from 2.1% to 11.8% (13), and our results were similar. In the ETAA group, a magnified view and intraoperative nerve monitoring assist in the identification and protection of RLN, which can reduce the incidence of injury (22). The incidence of transient hypoparathyroidism ranged from 1.2% to 40% (23); our results were 12.9% and 15.2% in the ETAA and OT group, respectively. A magnified view and the use of carbon nanoparticles are helpful in protecting the parathyroid *in situ*. However, it is difficult to retain the inferior parathyroid with type A by ETAA when cleaning the lower VI lymph nodes or superior parathyroid with types A2 and A3 because of the easily damaged blood supply (24). We implanted ischemic and resected parathyroid tissue in the sternocleidomastoid or deltoid in order to avoid transient hypocalcemia. According to the study by Kim et al. (25), parathyroid autotransplantation is an effective therapeutic option for permanent hypoparathyroidism, but we lack relevant experience. No patients in this study suffered from permanent RLN injury or hypoparathyroidism.

Choking cough was one of the clinical symptoms due to RLN or SLN injury, which occurred in one patient from the OT group. This patient drank at the proper angle by bowing their head and the symptom was relieved within 3 weeks. Furthermore, some patients complained of subtle voice change without abnormal laryngoscope outcomes, which may be attributed to the external branch of SLN injury. In fact, SLN injury, especially external branch injury (EBSLN), was underestimated clinically because postoperative laryngoscopy and voice analysis were not routinely used in our center. When accurate laryngeal electroneuromyography was used to diagnose abnormal conductivity, Cernea et al. indicated that the incidence of EBSLN was as high as 58% (26). It was clinically rare due to its technical difficulty and cost. Therefore, we were unable to evaluate the advantages or disadvantages of ETAA in protecting SLN. No infection occurred in patients of the ETAA group, possibly due to the class I incision and unobstructed drainage. During the hospital stay, none of the patients underwent postoperative hematoma and the second surgery. To sum up, ETAA is a safe surgical method with low complication rates for patients with PTNMC.

Compared with the OT group, the CRP level was higher in the ETAA group, possibly due to the long surgical time and flap trauma. In spite of the statistical difference in total drainage amount, drainage duration and postoperative hospital day were similar. The analgesic was not used routinely and the postoperative 24-h VAS scores did not differ significantly between the two groups. Thus, the increase of surgical trauma was mild and acceptable. Jiang et al. (27) considered that the dissection plane was the major factor leading to pain. Thus, to reduce postoperative discomfort, surgeons should have a more comprehensive understanding of the anatomy.

According to the national consensus and CACA guidelines (28), all patients underwent unilateral or bilateral central lymph node dissection. The number of dissected and positive lymph nodes plays a vital role in guiding postoperative treatment. In addition, for patients who underwent bilateral thyroidectomy, the postoperative serum Tg level and the proportion of sTg of <1 μg/L are crucial for evaluating the oncological completeness (29). There were no statistically significant differences in those indexes mentioned above and in persistence/recurrence. Thus, we confirmed that the oncological completeness was comparable between the ETAA technique and OT.

According to the ATA guideline (7), when the disease-free condition was maintained for at least 1 year, the occurrence of a new lesion indicated the recurrence. Conversely, the new lesion was defined as persistent disease. However, this definition is still controversial, and our study made no distinction. Persistence/recurrence were confirmed by puncture pathology in two patients of the ETAA group 6 months after surgery and one patient of the OT group 4 years after surgery. All of them were treated with a second open surgery on account of the increase of operative difficulties and risks. Wang et al. reported the feasibility of second surgery using ETAA (30). However, most of the patients (80.4%) were diagnosed with nodular goiter, and ETAA should be cautiously performed for patients with recurrence even though they are extremely worried about the scars. In a meta-analysis involving 9,369 patients with PTC, the rate of lymph node metastasis (LNM) ranged from 13.9% to 64.7% and tumor size >1 cm was one of the aggressive factors (31). In our study of PTNMC, the proportion of metastatic lymph nodes was 57.6% and 65.6% in the ETAA and OT group, respectively, which was consistent with previous research.

Cosmetic requirements promote rapid advances in endoscopic technology. A visible neck scar may increase the concerns of patients, especially among the Asian population (32). In this research, the patients in the ETAA group demonstrated a higher cosmetic satisfaction and QoL, in line with many previous reports. Nonetheless, three patients in the ETAA group complained of numbness in the chest area resulting from injury of the cutaneous nerve, which recovered within 3 months, but possibly decreased short-term QoL.

Because this was a single-center retrospective study, selection bias in surgical approach could not be avoided. In addition, only 151 matched pairs in this study were included and their follow-up time was short, which may reduce the credibility of the long-term oncology effect. Thus, larger samples and multicenter prospective studies with long-term follow-up are needed to confirm the safety, feasibility, and long-term oncological outcomes of ETAA in the treatment of PTNMC.

## Conclusion

ETAA produces similar surgical and oncological outcomes in treating PTNMC (<4 cm) compared with OT. Moreover, it provides better cosmetic outcomes despite a longer operative time and larger drainage. ETAA is a safe and feasible surgical approach for patients with PTNMC.

## Data availability statement

The original contributions presented in the study are included in the article/supplementary material. Further inquiries can be directed to the corresponding author.

## Ethics statement

This study has been approved by The Ethical Committee of the Second Affiliated Hospital of Zhejiang University’s School of Medicine. The patients/participants provided their written informed consent to participate in this study.

## Author contributions

Study design: ZL, YW, and PW. Data collection: YL, ZL, and ZS. Data analysis: YL and XY. Drafting the manuscript: YL. Project supervision: YW. All authors contributed to the article and approved the submitted version.
